# Mechanisms that limit proliferative potential of drug-specific LTT in drug-induced severe cutaneous adverse reaction patients

**DOI:** 10.1186/2045-7022-4-S3-O1

**Published:** 2014-07-18

**Authors:** Laurence Valeyrie-Allanore, Maja Mockenhaupt, Peggy Sekula, François Berrehar, Nicolas Ortonne, Haudrey Assier, Colette Goujon, Martine Bagot, Jean-Claude Roujeau, Armand Bensussan, Sabine Le Gouvello

**Affiliations:** 1 Henri Mondor University Hospital, RegiSCAR France & Dermatology Department, France; 2 Freiburg University Medical Center, RegiSCAR & Dermatology Department, Germany; 3 Freiburg University Medical Center, Epidemiology and Statistics Department, Germany; 4 Henri Mondor University Hospital, ImmunoBiology Department, France; 5 Henri Mondor University Hospital, Pathology Department, France; 6 Henri Mondor University Hospital, Dermatology Department, France; 7 Henri Mondor University Hospital, Neurology Department, France; 8 University Medical Center Saint-Louis, Dermatology Department, France; 9 University Medical Center Saint-Louis, Inserm U976, France; 10 University Medical Center Henri Mondor, ImmunoBiology Department & Inserm U955, France

## Background / Objective

Prior use of "lymphocyte transformation test" (LTT) suggested that it was less often positive in Stevens-Johnson syndrome (SJS), Toxic Epidermal Necrolysis (TEN), than in other cutaneous reactions, with possible dependence on sampling date. We explored the possible role of inhibitory co-receptors in LTT, using well-defined groups of patients who reacted to carbamazepine (CBZ), or lamotrigine (LTG), sulfamethoxazole (SMX) and allopurinol (ALL).

## Method

Thirty one cases of SJS/TEN, and controls patients with DRESS (21) or exposed without reaction (EWR; (20)) were provided by the RegiSCAR group. Peripheral mononuclear cells (PBMC) were tested by qRT-PCR for the expression of the inhibitory co-receptors and their respective ligands : PD-1/PDL1, CTLA4/B7.1. LTT was performed with PBMCs by measuring H3-thymidine incorporation after 6 days incubation with CBZ/LTG/ALL (10µg/ml), SMX (50µg/ml) or medium, in the presence or not of blocking antibodies. Stimulation index ≥ 2.5 was considered positive. Mann-Whitney U test was used for comparison of gene expression level. A p value ≤0.05 was considered statistically significant.

## Results

Positive LTT was observed in 3/23 (13%) SJS/TEN and in 2/18 (11%) DRESS tested during the acute phase, and 7/22 (32%) SJS/TEN and 4/12 (33%) DRESS tested after recovery (late). LTT were all negative in EWR. As compared to their expression in PBMC of EWR, i) overexpression of CTLA4 and of B7.1 was found in acute and late SJS/TEN, respectively; ii) overexpression of PD-1 and PDL1 was found in acute DRESS. Combined addition of anti-CTLA4 and anti-PDL1 mAbs to LTT cultures of SJS/TEN (2 CBZ and 2 SMX) and DRESS (4 CBZ) increased drug-induced proliferation index, even turning some negative LTT into positive LTT (4 out of 8; 50%).

## Conclusion

We confirm that reactive T cells are rarely detected in acute phase of SCAR, and to a lesser extent after recovery. We show for the first time that CTLA4- and PD-1 pathways are active in SJS/TEN and DRESS, respectively, and may contribute to the negative reactivity of LTT. The use of anti-CTLA4 and anti-PDL1 mAbs could help to sensitize drug-specific LTT.

